# An Evaluation of the Efficacy of Four Currently Used Sheep Pox Vaccines Against a Contemporary Virulent Strain

**DOI:** 10.3390/vaccines13121243

**Published:** 2025-12-15

**Authors:** Irina Shumilova, Mohammad Abed Alhussen, Alena Krotova, Kseniya Shalina, Pavel Prutnikov, Svetlana Kononova, Olga Byadovskaya, Ilya Chvala, Larisa Prokhvatilova, Alexander Sprygin

**Affiliations:** Federal Center for Animal Health, 600901 Vladimir, Russiaalhussenmohammed85@hotmail.com (M.A.A.);

**Keywords:** sheep pox, Capripoxvirus, challenge, RM65 strain, KSGP 0240 strain, KSGP ARRIAH strain, NISKHI ARRIAH strain

## Abstract

Background/Objectives: Sheep pox, a highly contagious disease, is prevalent in Africa and Asia, with sporadic outbreaks in Europe, and inflicts tremendous economic losses. Vaccination represents the primary and most effective prevention method. The genetic diversity of circulating SPPV strains worldwide is poorly studied, and vaccine selection is typically guided by the availability of a particular vaccine. In this study, four sheep pox vaccines, including the RM65, KSGP 0240, KSGP ARRIAH, and NISKHI ARRIAH vaccines, were evaluated against a contemporary virulent strain circulating in Asia. Methods: The level of antibodies in the blood serum was determined using the ELISA and microneutralization assay. Blood samples and nasal swabs were obtained for PCR examination. Comprehensive clinical and postmortem pathological examinations were conducted. Results: The body temperature of all experimental animals remained within the physiological norm, with no clinical manifestations, local reactions, viremia, or necropsy pathological lesions, demonstrating the effectiveness and safety of the vaccines used against the contemporary virulent strain. Furthermore, immunization was associated with the formation of neutralizing and specific antibodies in all vaccinated groups post vaccination, with a significant increase in their levels after challenge, indicating a high level of immunogenicity. The NISKHI ARRIAH vaccine exhibited statistically significant superiority over the other vaccinated groups. However, the unvaccinated control group demonstrated post-challenge moderate-to-severe clinical signs, postmortem lesions, with high levels of virus shedding, and lower levels of neutralizing and specific antibodies, compared with the vaccinated groups. Conclusions: Our study results indicate that the experimental group immunized with the NISKHI ARRIAH vaccine exhibited the initial and most substantial immune response, maintaining the highest antibody levels on the 28th day after vaccination in comparison to the other studied vaccines.

## 1. Introduction

The genus Capripoxvirus (CaPV) comprises three species: lumpy skin disease virus (LSDV), sheep pox virus (SPPV), and goat pox virus (GTPV) [1,2]. These species share double-stranded DNA genomes of approximately 150 kbp each and exhibit 96% nucleotide identity between the three species within the genus [3,4]. The CaPV genome encodes 147 putative genes; genes responsible for replicative mechanisms are located in the central core region, whereas genes that mediate pathogenesis and host range functions are positioned at termini [3,5].

Sheep pox and goat pox, caused by SPPV and GTPV, respectively, were previously considered to be a single disease until genome sequences revealed the differences between the SPPV and GTPV strains [6]. Notwithstanding the stringent host specificity characteristic of the Poxviridae family, SPPV and GTPV do not align with other single-host-restricted poxviruses and can cross-infect both sheep and goats [2,7]. There is evidence indicating that sheep strains cause mild disease in goats and severe disease in sheep, whereas virulent goat strains can cause mild disease in sheep depending probably on the host genetics background [2,8,9].

The diseases caused by the CaPV genus are characterized by a variety of clinical manifestations, including fever, depression, diarrhea, rhinitis, coughing, conjunctivitis, and other symptoms [10,11,12]. Furthermore, the affected animals typically develop characteristic lesions, represented by papules and nodules in the skin [10,12,13,14]. The aforementioned lesions can be localized, restricted, affecting hairless areas [10], or generalized [11,12,13,15]. SPPV transmission occurs primarily through direct contact and aerosols and indirectly via contaminated objects, feed, wool, and contamination of abrasions and wounds; moreover, it has been established that insects play a minor role in transmission (mechanical vectors) [12,15,16,17].

SPPV and GTPV inflict significant economic burdens due to the high morbidity and mortality rates of the affected hosts. In the context of an outbreak, SPPV can wipe out almost an entire population, with morbidity reaching up to 100% and mortality reaching up to 85% [2,16,18]. CaPV infections in small ruminants exhibit a global distribution and are spread in areas where people depend on sheep for livelihood [2]. These geographical regions include Africa, the Middle East, and Asia. Recent outbreaks in northern latitudes in Eurasia indicate a pronounced tendency for range expansion into naïve ruminant populations [2,10,14].

Vaccination has been recognized as an efficient tool in curbing outbreaks and eradicating the disease. The available vaccines are either live-attenuated or inactivated [11,19,20]. The live-attenuated vaccine confers robust, lifelong protection, serving as the optimal long-term solution in endemic areas, while inactivated preparations do not offer protracted immunity and require two doses, which can be attributed to cell-mediated immunity playing the principal role in long-term immunity; however, the inactivated vaccine can be used in non-endemic countries in emergency scenarios and in countries where the use of live vaccines is unauthorized [11,17,19,20].

Currently, several SPPV vaccines are used in various countries. In India, the Romanian Fenner and Srinagar vaccine strains are employed [21,22,23,24]; in Turkey, sheep vaccination is executed utilizing the indigenous Bakırköy strain [25], whereas the Romanian and Yugoslavian RM65 SPPV vaccine strains are utilized in Morocco, Egypt, Senegal, and Algeria [24,26]. The NISKHI SPPV vaccine strain is employed in Russia, Kazakhstan, and other ex-Soviet Union countries to vaccinate sheep, goats, and cattle [27,28,29,30]. In Ethiopia, annual mass vaccination is conducted using a heterologous strain of the Kenyan sheep and goat pox (KSGP) O-180 virus to immunize small ruminants against sheep and goat pox and to protect cattle against LSDV [2,31,32]. The aforementioned vaccines demonstrate good protection profiles; however, there has been no attempt to assess them against each other in a broader context [33]. The literature search could not identify studies pertaining to the direct comparison of currently used vaccine strains in a controlled setting against contemporary isolates.

Notwithstanding the implementation of vaccination programs, SPPV can still cause outbreaks, even among sheep flocks vaccinated with RM65 in Algeria or with KSGP in Ethiopia, which are endemic countries [31,34]. Furthermore, sporadic incursions have been documented in SPPV-free regions [27,35]. To elucidate the reasons underlying the recurrent outbreaks in a vaccinated context, given the worldwide availability of vaccines and the existence of virulent challenge strains, further comprehensive studies are required to inform sheep pox management campaigns [34]. However, given their experience, the authors strongly recommend a challenge study to guide the vaccine strategy in a given region [34]. This approach appears to be both logical and reasonable, as such a study would provide scientifically substantiated findings to guide vaccine selection.

The objective of this study was to compare the protective capacity of the RM65, KSGP 0240, KSGP ARRIAH, and NISKHI ARRIAH vaccines in experimental sheep against a virulent contemporary field strain.

## 2. Materials and Methods

### 2.1. Vaccines

The following vaccines were evaluated: the RM65 strain vaccine, the KSGP 0240 strain vaccine, the KSGP ARRIAH strain vaccine, and the NISKHI ARRIAH strain vaccine. The vaccines were confirmed to be free of contaminants, assessed for purity, and identified according to OIE Terrestrial Code, 2018 [36]. The virus titer in a dose of 1.0 mL of each of the lyophilized vaccines was not less than 10^3.0^ TCID_50_/mL. The vaccines were reconstituted with sterile phosphate-buffered saline (PBS) according to the manufacturer’s recommendations and utilized immediately.

### 2.2. Challenge Virus

A virulent SPPV isolate was obtained from a sheep pox outbreak in Russia in 2023 and was assigned to cluster 1.1 [37]. The isolate was propagated on goat gonad cells for several passages, reaching a titer of 10^5.3^ TCID_50_/mL. Thereafter, it was confirmed to be free of contaminants and assessed for purity. The challenge was executed by subcutaneous injection of 2 mL of the virulent SPPV isolate on the 21st day post vaccination.

### 2.3. Experimental Design

Twenty-five sheep heads of the Romanian breed, aged 6–9 months, with different genders, unvaccinated against sheep pox or goat pox, were randomly allocated into five homogenous groups, with five animals per group, as follows:

Experimental group 1 (animals numbered 1–5) received the RM65 strain vaccine (undisclosed manufacturer).

Experimental group 2 (animals numbered 6–10) was administered the KSGP 0240 strain vaccine (undisclosed manufacturer).

Experimental group 3 (animals numbered 11–15) received the KSGP vaccine (FGBI ARRIAH, Russia).

Experimental group 4 (animals numbered 16–20) was administered the NISKHI vaccine (FGBI ARRIAH, Russia).

The control group (animal numbered 21–25), unvaccinated animals, received a mock vaccination, consisting of a reconstitution buffer.

The nomenclature of the vaccine was assigned based on the strain contained therein.

Animals were allowed to acclimatize for a 21-day period prior to experiment initiation, with daily monitoring for the appearance of nonspecific clinical signs, such as fever, salivation, and other symptoms. Animals were fed a forage-based diet with free access to water.

Prior to vaccination, samples of stabilized blood and nasal swabs were collected for PCR examination to confirm the absence of sheep pox, goat pox, or other pathogens. Furthermore, blood serum samples were collected from all animals to determine the initial level of antibodies to Capripoxvirus (zero background).

Each group was housed in a separate room under biosafety level 3 (ABSL-3) isolation conditions. The animals of the experimental groups were administered 2 mL of the appropriate vaccine subcutaneously in the woolless area of the right armpit. All animals were subjected to observation, including the measurement of body temperature, clinical and vital sign monitoring, and site of injection reaction measurement, until the end of the experiment (28 days post vaccination; 7 days post infection). At the end of the experiment, the control and experimental animals were euthanized and underwent subsequent pathological examination.

### 2.4. PCR Testing

Samples were collected from all animals for a period of 7 days post vaccination, with a 1-day interval. Beginning on the 8th day, samples were collected at 3-day intervals. Post challenge, samples were periodically collected for a period of 14 days (22–35 days of the experiment) from all animals. Blood samples and nasal swabs were collected aseptically, according to a previously published method [38]. Total nucleic acid was extracted following a previously established phenol–chloroform extraction protocol [39]. Extracts were then subjected to real-time PCR (qPCR) using primers directed against RPO132, as previously described [40]. Briefly, PCR was performed in a Rotor-Gene Q5 plex HRM thermocycler (QIAGEN, Hilden, Germany) using a commercial SsoFast™ EvaGreen^®^ Supermix kit (Bio-Rad Laboratories—Hercules, CA, USA) with an initial denaturation stage at 98 °C for 2 min, followed by 40 cycles at 95 °C for 5 s and 60 °C for 20 s. The PCR product was then denatured at 95 °C (30 c exposure), cooled to 65 °C (60 c exposure), and melted from 65 °C to 90 °C with a temperature increase of 0.1 °C every 2 s with continuous data collection. The amplification plots and melting graphs were analyzed using the Rotor-Gene Q5 plex, and the corresponding curves were displayed as negative first-derivative plots of fluorescence with respect to temperature. Normalized melt curves and differences in curves were acquired by analyzing the active melt region separately for each virus by designating the corresponding pre- and post-melt regions [40].

### 2.5. ELISA Testing

Blood samples were collected weekly on days 0, 7, 14, 21, and 28 of the experiment; samples were centrifuged at 3000× *g* for 10 min, and the serum was harvested and stored at a temperature of −80 °C until use. To detect the specific antibodies of the Capripoxvirus in the serum or plasma of susceptible animals, the ELISA test system “ID Screen^®^ Capripox Double Antigen” Multi-species ID.VET” (ID. Vet, France) was used according to the manufacturer’s instructions. Interpretation of the results was conducted as aggregate to the positive control ratio S/P percentage (S/P %) using the following formula:

$$S/P\;\%\;=\;\frac{sample\;OD-negative\;control\;OD}{positive\;control\;OD-negative\;control\;OD}\;\times \;100$$

OD: optical density.

The ELISA results were interpreted as follows: S/P % ≥ 30%—positive; S/P % ˂ 30—negative.

### 2.6. Microneutralization Assay

The presence of virus-neutralizing antibodies in the serum samples was detected using a microneutralization (MN) assay in a sensitive subculture of lamb testicular cells (LTCs). Two-fold dilutions of the test sera were prepared in a 96-well cell culture plate (JetBioFil, Guangzhou, China) at a volume of 50 μL, with an equal volume of virus-containing culture material (the “ARRIAH” SPPV strain, 100 TCID_50_/mL). The plates were incubated in a 5% CO_2_ incubator at a temperature of 37.0 ± 0.5 °C for 1 h. Then, 0.1 mL of cell culture suspension containing 0.4–0.5 × 10^6^ cells/mL was added to all wells and incubated in 5% CO_2_ at a temperature of 37.0 ± 0.5 °C for 96–120 h. Observations were carried out using an invertible microscope at 400× magnification, and wells with a pronounced viral CDP and/or with a single intact monolayer were registered. The results of the dilution tests were considered positive at a ratio of <1:8, doubtful at a ratio of <1:4, and negative at a ratio of <1:2. Subsequently, the neutralizing antibody titer was expressed in log_2_.

### 2.7. Statistical Analysis

The statistical analysis was performed using the IBM SPSS Statistics (version 26.0) software. The mean of the neutralizing antibody titers (log_2_) and the SP% ratio for each time point were analyzed independently using one-way Analysis of Variance (ANOVA) to test for an overall significant difference among the five tested groups. When the ANOVA indicated a significant overall difference (*p* < 0.05), Tukey’s Honestly Significant Difference (HSD) post hoc test was applied to perform pairwise comparisons between all groups, including the experimental groups compared to the control group and the experimental groups compared to each other. A statistical significance level of α = 0.05 was used for all tests.

### 2.8. Ethics Statement

The experiments were conducted in accordance with the guidelines of the European Union Council (Directive 2010/63/EU). The study was approved by the Ethics Committee of the Federal Center for Animal Health (FGBI “ARRIAH”), Russia (permit number: No. 4/32-12122024). To alleviate their suffering, animals were stunned using captive bolt penetration, followed by the intravenous injection of a muscle relaxant, Adilin Super (Federal Center for Toxicological, Radiation and Biological Safety, Kazan, Russia), at a dose of 5 mg/kg, following the manufacturer’s instructions.

## 3. Results

### 3.1. Clinical Evaluation of Animals

Following vaccination, the body temperature of all experimental animals remained within the physiological norm; no clinical signs or observable changes were detected at the vaccine injection site in any group. Concurrently, the aforementioned parameters in the control group animals were found to be normal.

**Figure 1:**
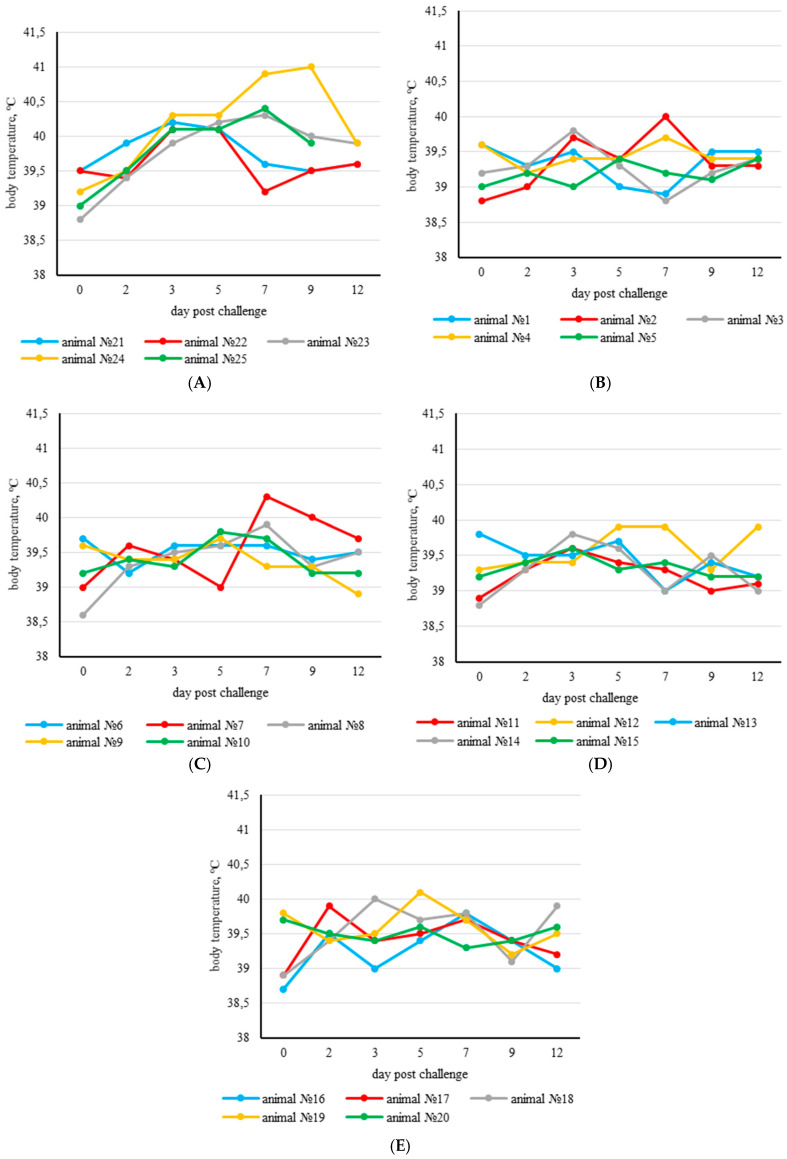
Body temperature dynamics in animals of the control and experimental groups after challenge. Increased body temperature was defined as >40.0 °C. (**A**) Control group; (**B**) experimental group 1 (the RM65 strain vaccine); (**C**) experimental group 2 (the KSGP 0240 strain vaccine); (**D**) experimental group 3 (the KSGP ARRIAH strain vaccine); (**E**) experimental group 4 (the NISKHI ARRIAH strain vaccine).

After the challenge with a virulent SPPV isolate, the physiological parameters of the experimental vaccinated animals were within normal limits. The body temperature of the majority of the experimental animals remained within the normal range until the end of the experiment. However, a transient mild rise in body temperature was recorded in animal №.7 on the 7th day (G2 KSGP 0240) and in animal №.19 (G4 NISKHI ARRIAH) on the 5th day post challenge (Figure 1B–E).

**Figure 2:**
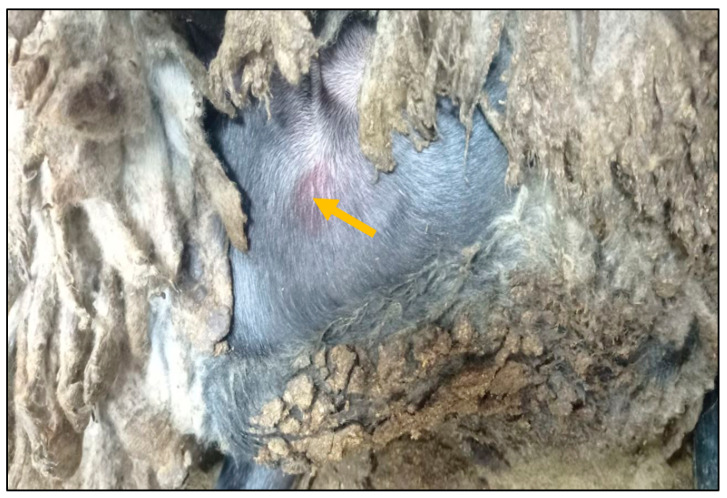
Swelling and erythema at the injection site of the virulent sheep pox virus (animal №.4).

It is noteworthy that on the 5th day following the challenge, the experimental animals exhibited local erythema and developed swellings at the injection site measuring between 0.5 and 4 cm in diameter (Figure 2). These changes were observed for three days and subsequently disappeared by the 7th day.

**Figure 3:**
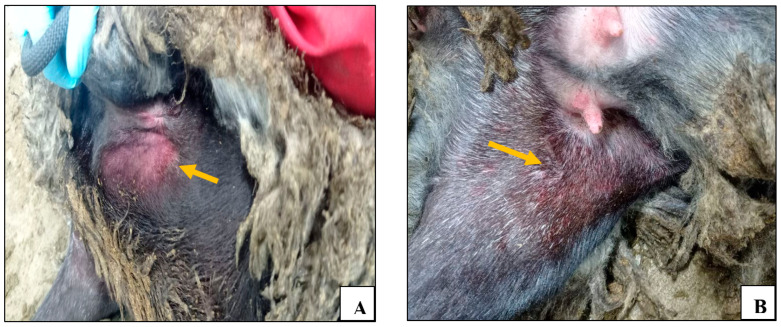

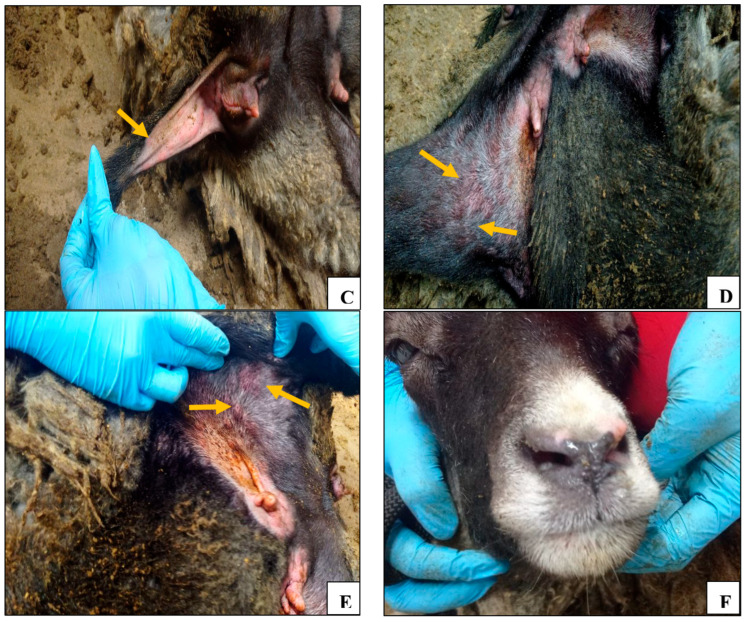
Clinical signs in control animals after infection with the virulent sheep pox virus: (**A**) erythema and swelling at the virus administration site (animal №.22); (**B**) confluent lesions on the inner thigh (animal №.25); (**C**) roseola in the perineum (animal №.25); (**D**) roseola in the groin area (animal №.25); (**E**) roseola in the groin area (animal №.23); and (**F**) nasal discharge (animal №.23).

As for the control unvaccinated group, beginning on the 3rd day post challenge, the control group animals demonstrated a significant increase in body temperature (Figure 1A), lethargy, erythema, and swelling at the site of virus administration (Figure 3A). Furthermore, signs of nasal discharge, cough, rhinitis, and conjunctivitis were observed in animals №.23 and 24 (Figure 3F). Additionally, animals №.23 and 25 developed roseola in the groin area (Figure 3B–E).

On the 12th day post challenge, animal №.25 was removed from the experiment due to death. This animal had previously exhibited characteristic symptoms of sheep pox, manifesting as generalized pustular lesions in hairless areas of the body (axillae, groin) (Figure 3B–D).

**Figure 4:**
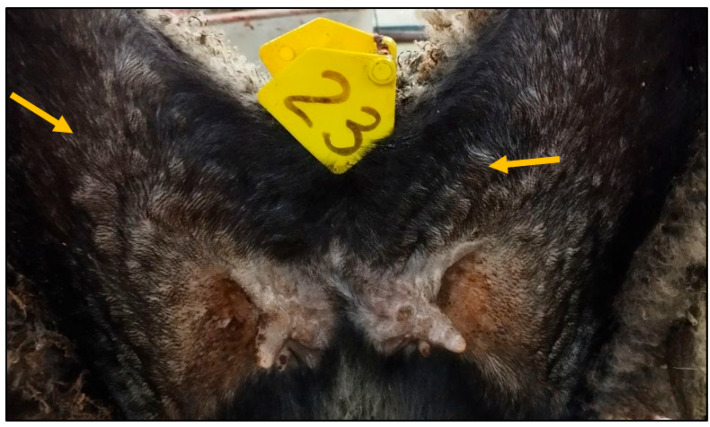
Pustular rash in the groin and inner thigh area (animal №.23).

The postmortem examination revealed the presence of a pustular rash in animal №.23 from the control group (Figure 4); however, no such pustular rash was detected in the other control animals.

### 3.2. Gross Lesions

**Figure 5:**
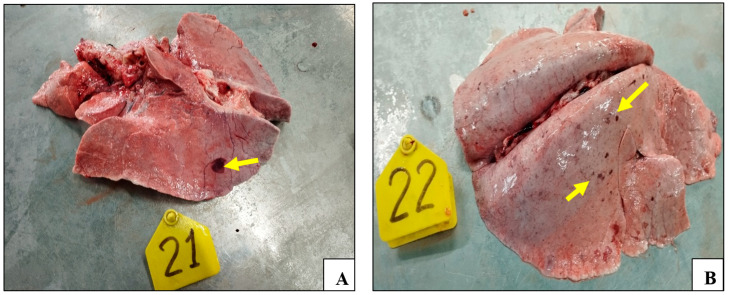

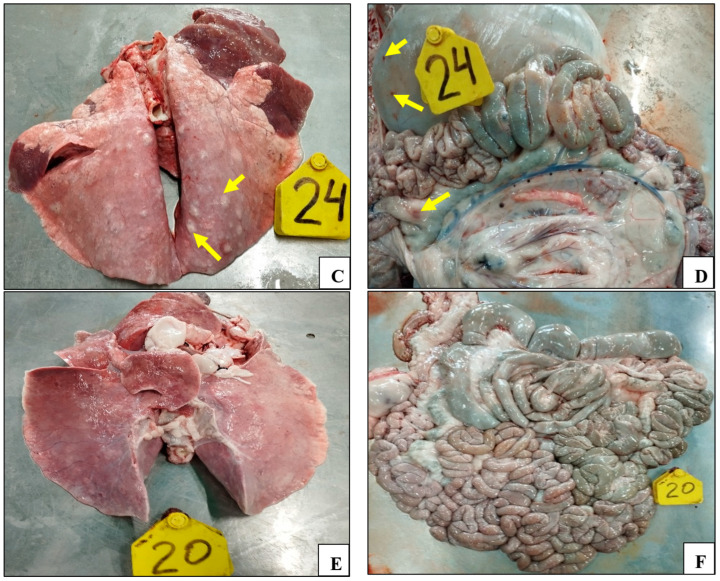
Smallpox lesions observed in organs of the control animals post challenge with the virulent sheep pox virus: (**A**) lungs (animal №.21); (**B**) lungs (animal №.22); (**C**) lungs (animal №.24); (**D**) hemorrhages on the gastrointestinal tract surface (animal №.24); (**E**) lungs of vaccinated animal (animal №.20); and (**F**) gastrointestinal tract surface of vaccinated animal (animal №.20).

The necropsy results demonstrated the presence of lesions in the lungs of all of the control animals. The lungs of these animals demonstrated signs of pathoanatomical changes, including enlargement, edema, and multiple focal hemorrhages on all surfaces of the lungs (Figure 5A–C).

Furthermore, sheep pox lesions were identified on the serous membrane of the abomasum in animal №.24; single and multiple hemorrhages were found on the entire intestinal surface (Figure 5D). On the contrary, no pathoanatomic changes were detected in the animals of the experimental groups (Figure 5E,F).

### 3.3. Viral DNA Detection

Prior to the initiation of the experiment and the post-vaccination period, the qPCR results showed that the genome of the SPPV was not detected in the blood samples or nasal swabs obtained from both the control and vaccinated experimental animals. Following the challenge, the qPCR results were negative in the animals of the experimental groups until the end of the experiment. The qPCR results of the control group animals after the challenge are shown in Table 1.

As presented in Table 1, sheep pox genomic DNA was detected in the unvaccinated control animals challenged with the SPPV inoculum, as confirmed by the positive qPCR results in their nasal swab and/or blood samples. Viral genomic DNA was identified in the nasal swabs of animals №.21–22 from days 7 to 12 post infection. However, no detectable DNA was identified in the blood samples from animal №.22, whereas in the blood samples of animal №.21, DNA was detected only on the 5th day after the challenge. The viral genome in the other control animals (№.23, 24, and 25) was detected in the nasal swabs and blood samples from the 5th day and continued to be shed until the end of the experiment.

### 3.4. Neutralizing Antibody Titers

**Figure 6:**
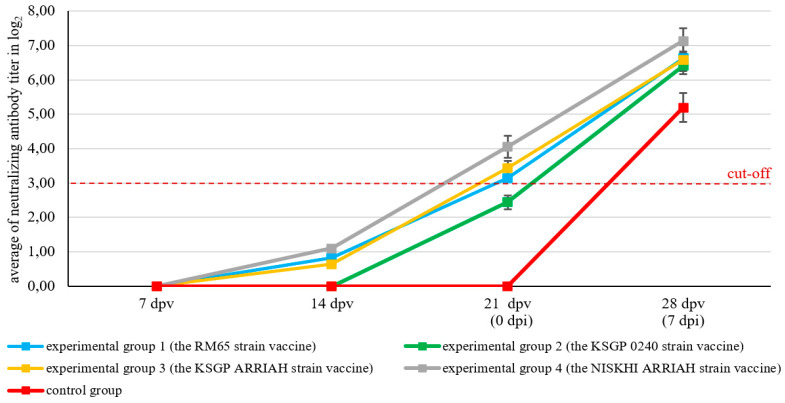
Neutralizing SPPV antibody kinetics in experimental and control animals determined by microneutralization assay (mean of neutralizing antibody titer ± SE). dpv: day post vaccination; dpi: day post infection. Interpretation of results: average antibody titer in log_2_ ≥ 3—positive; log_2_ ˂ 3—negative.

Our study results indicated that immunization with various SPPV vaccines was accompanied by the formation of virus-neutralizing antibodies against SPPV in the serum of the experimental groups. By the 14th day post vaccination, significant differences in mean the log_2_ titer were observed between the study groups (ANOVA, *p* < 0.0001). Groups 1, 3, and 4 (RM65, KSGP ARRIAH, and NISKHI ARRIH) showed detectable mean antibody titers of 0.82 ± 0.07, 0.64 ± 0.04, and 1.10 ± 0.04 log_2_, respectively, compared to group 2 (KSGP 0240) and the control group, which demonstrated no alteration in the titer of neutralizing antibodies. However, all groups remained below the seropositive threshold of log_2_ ≥ 3. On the 21st day after vaccination, an increase in the mean antibody titers, ranging from 2.44 ± 0.20 to 4.05 ± 0.32 log_2_, was recorded in the animals of all experimental groups; the overall effect was highly significant among the groups (ANOVA, *p* < 0.0001). By this time, the fourth experimental group (NISKHI ARRIH) had achieved the highest mean neutralizing titer (4.05 ± 0.32 log_2_), demonstrating the most robust pre-challenge immunity. Furthermore, Tukey’s HSD test confirmed the statistical superiority of the fourth group (NISKHI ARRIH) (*p* < 0.0001). In contrast, the second experimental group (KSGP 0240) failed to seroconvert with a mean titer of 2.44 ± 0.20 log_2_. On the 28th day after vaccination, the mean antibody titers were significantly higher in the experimental groups compared to the control group (ANOVA, *p* = 0.0088), ranging from 6.41 ± 0.25 to 7.14 ± 0.37 log_2_. Notably, the highest titers were observed in the first (RM65) and fourth (NISKHI ARRIAH) groups (*p* = 0.0048) (Figure 6), with a statistically superior result for the fourth group (NISKHI ARRIAH) (*p* < 0.05). As anticipated, no increase in the antibody titer levels was observed in the blood serum of control animals until the 21st day after mock vaccination, and by the 28th day, their mean value was 5.20 ± 0.42 log_2_ (Figure 6).

### 3.5. Antibody Levels

The microneutralization reaction results were consistent with those of the ELISA, wherein the initial seropositivity results (seroconversion) were detected on the 21st day post vaccination (challenge day) in all of the experimental animals. A highly significant overall difference was found between the groups for all time points (ANOVA, *p* < 0.05). The S/P % ratios for all experimental groups on the 21st day post vaccination ranged from 37.8 ± 0.26 to 44.00 ± 0.40, with highly significant antibody responses (*p* ≤ 0.001) compared to the control group. The control group remained seronegative, with a mean S/P % ratio of 22.0 ± 0.89. The fourth experimental group (NISKHI ARRIAH) showed significantly higher antibody levels compared to the other experimental groups (*p* ≤ 0.05) on the 21st day post vaccination. However, no significant differences were observed among the first, second, and third experimental groups (RM65, KSGP 0240, and KSGP ARRIAH).

**Figure 7:**
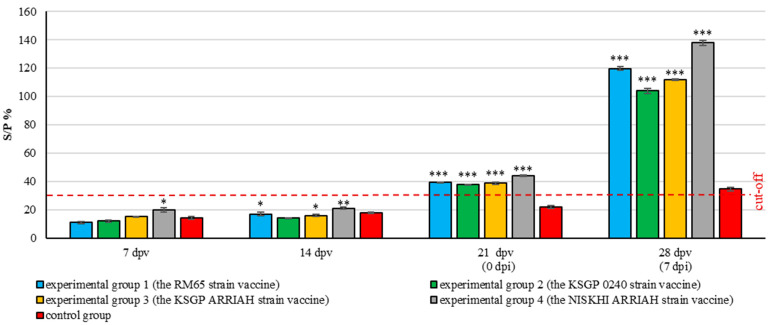
The serological examination results for SPPV antibodies in experimental and control groups determined by ELISA (mean ± SE). dpv: day post vaccination; dpi: day post infection. Interpretation of results: S/P % ratio ≥ 30%—positive; S/P % ratio ˂ 30—negative. * *p* < 0.05, ** *p* < 0.01, and *** *p* < 0.001 compared to the control group.

The S/P % ratio for all vaccinated groups continued to increase after the challenge, reaching a peak on the 28th day post vaccination (7th day post challenge), with a highly significant difference compared to the control group (*p* ≤ 0.001) (Figure 7).

It is noteworthy that the fourth experimental group (NISKHI ARRIAH) exhibited the earliest significant immune response (7th day) and maintained the highest antibody levels on the 28th day after vaccination compared to the other experimental groups (*p* ≤ 0.001). This suggests that the vaccine elicited the most potent and prolonged antibody response, both prior to and following the challenge. The response of the first experimental group (RM65) was found to be significantly higher than that of the second and third groups (*p* ≤ 0.001) (KSGP 0240 and KSGP ARRIAH) on the 28th day after vaccination, with no significant difference found between the third and second groups (KSGP 0240 and KSGP ARRIAH).

In contrast, the unvaccinated control group remained negative until the 28th day post vaccination (7th day post challenge), at which point, it slightly crossed the seropositivity threshold (mean SP% ratio of 34.6 ± 1.44), demonstrating a primary immune response to the infection; however, this level was significantly lower compared to those of all vaccinated groups.

## 4. Discussion

Sheep pox, a highly contagious and economically significant disease affecting small ruminants, can be controlled through the implementation of mass vaccination programs utilizing potent vaccines [2,41,42]. Many vaccines have been developed against sheep pox in countries where ruminants are at a risk of contracting a Capripoxvirus disease. Vaccines can be either live or inactivated and homologous or heterologous and possess varying safety and efficacy profiles [13,28,43].

The necessity for a highly effective, safe, and locally available sheep pox vaccine is imperative, as imported exotic strains of attenuated vaccines have demonstrated insufficient effectiveness under local conditions, and there is inadequate safety data for the utilization of indigenous SPPV strains in the field [21,22]. This entails a comparison of the currently employed vaccines with the locally developed vaccine, in order to assess their safety, potency, and protective efficacy against local virulent virus strains [21]. This methodology will facilitate the selection of a suitable and cost-effective vaccine for mass immunization, in addition to the utilization of viruses with a restricted host range, which are well-adapted to the specific local strains that are responsible for the manifestation of the disease [16,21,22].

A review of the scientific literature revealed only a few recent publications that directly compared the efficacy of different vaccines in a controlled environment using a contemporary virulent strain of the sheep pox virus [11]. In one such study, conducted by Algerian scientists, the vaccine strains Djelfa, Romania, Istanbul, and KSGP were compared against a local virulent SPPV strain (genetic affiliation undetermined). The results indicated that the Djelfa strain conferred good immunity to animals without adverse effects post vaccination, and the Romania strain provided prolonged protection beyond twenty-four months but with undesirable persistent vaccine adverse reactions, compared with the Istanbul and RM65 strains, which demonstrated insufficient protection [44]. Conversely, the Rm-65 strain was found to be superior to the Romanian strain when evaluated under field conditions in Egypt as part of emergency vaccination [20].

In this work, the efficacy of four sheep pox vaccines, including the RM65, KSGP 0240, KSGP ARRIAH, and NISKHI ARRIAH strains of various manufacturers, was evaluated against a virulent SPPV isolate circulating in the region [27].

Notably, the NISKHI vaccine proved to be the most efficacious against a virulent isolate from cluster 1.1, found to be unique to the region [27]. Given that the original strain NISKHI was derived from a circulating field isolate in the region, thereby representing a shared phylogenetic relationship, it can be posited that all isolates from the region are distantly related within the group, which has a positive effect on the protection conferred [27].

Following the administration of the vaccines, no clinical signs or adverse reactions, manifesting as papules or lesions, were observed in any of the vaccinated animals. Furthermore, the average body temperature of all vaccinated animals remained within the physiological range (38.5–39.7 °C), and the genome of the SPPV vaccines was not detected in blood and nasal samples. These findings are consistent with those of previous studies [20,21,28,45]. In contrast, Achour et al. reported the appearance of vaccine-associated papules that developed into persistent lesions filled with fluid following RM65 vaccination [44]. On the other hand, the Romanian strain and other strains did elicit a transient increase in mean body temperature within a week of vaccine inoculation [46,47]; this phenomenon may be attributed to a typical feature of all live-attenuated vaccines, which may occur once administered [21,48].

The vaccines’ potency was evaluated against a virulent strain of cluster 1.1, representatives of which had never been biologically evaluated [37]. This lineage is unique to the region of post-Soviet Union space, and no information is available regarding its biological characteristics; however, severe virulence in field conditions has been reported. In the extant literature, vaccine strain assessments were performed with strains of unknown phylogenetic affiliation [11,21,44]; this represents a key limitation that precludes cross-study comparison of field isolate virulence. A few published studies evaluate challenge strains with complementary WGS data [12,28], including the present study.

In our study, the virulence profile was reproduced in control animals and was considered as a suitable challenge model with the new isolate representing cluster 1.1. All vaccinated groups remained clinically asymptomatic post challenge and showed negative real-time PCR results in their blood samples and nasal swabs, thereby confirming that the RM65, KSGP 0240, KSGP ARRIAH, and NISKHI ARRIAH vaccines induced immune protection without adverse reactions, with NISKHI ARRIAH achieving higher protection titers (*p* < 0.001). In addition, following the challenge with the virulent SPPV strain, the vaccinated groups showed local reactions persisting for three days (Figure 2) which disappeared by day 7 post infection. Analogous results have been published for bivalent vaccines comprising sheep pox and PPR in sheep [49] and goat pox and the PPR vaccine in goats [50].

The unvaccinated control animals demonstrated typical clinical signs of SPPV infection, manifesting as fever onset within three days after the challenge, which is in accordance with previously reported findings [11,51,52,53]. The maximum recorded body temperature was 41 °C, a finding that aligns with the results of Hamdi et al., following a challenge infection of unvaccinated sheep with the Turkish virulent SPPV Held strain [53]. Signs of anorexia, lethargy, erythema, and swelling at the site of virus administration were observed, consistent with previous observations describing the onset of clinical signs on around the 5th dpi [52]. In addition, nasal discharge, cough, rhinitis, roseola in the groin area, conjunctivitis, and generalized pustular lesions in hairless areas of the body were observed (Figure 3), characteristics of infections with virulent SPPV strains [11,51,52,53,54].

The unvaccinated control animals exhibited signs of edema, congestion, and hemorrhages in their lungs, as well as serosal and mucosal congestion of the gastrointestinal tract surface post challenge (Figure 5). These findings are consistent with those reported in previous studies [15,55,56].

Our study results indicated that viremia was detected on the 5th day post challenge (Table 1). Similarly, Bowden et al. detected Capripoxvirus DNA in blood samples on the 6th day post infection (dpi) in sheep experimentally infected with a Nigerian SPPV strain (10^4.9^ TCID_50_/mL) [51], whereas Babiuk et al. found the first positive PCR results in the blood of sheep experimentally infected with a Yemen Capripoxvirus isolate on the 8th dpi (10^4.7^ TCID_50_/mL) [54]. Furthermore, the positive PCR results of the nasal swabs indicate the robust replication of SPPV in the respiratory tract [12,51].

The results of this study revealed that neutralizing and specific antibodies were not detected in either the microneutralization assay or ELISA, respectively, for any of the vaccinated groups until the 14th day post vaccination, and by the 21st day after vaccination, the antibody response steadily increased. Moreover, the immune response was found to be boosted following the challenge, which indicates that all animals in all vaccinated groups were protected and able to produce antibodies in response to vaccine strains, signifying that the specific immune defenses had been efficiently induced, a finding that is consistent with the results of other studies [21,57,58]. In contrast, the control animals exhibited a slight increase in the antibody titer on the 28th day post vaccination. These findings indicate that the challenge virus elicits an immune response after causing severe disease in control animals [21].

Overall, our results indicate that the fourth experimental group vaccinated with the NISKHI ARRIAH strain exhibited the initial and most substantial immune response, maintaining the highest antibody levels on the 28th day after vaccination (SP% = 137.8 ± 1.78), compared to the other experimental groups (SP% = 119.8 ± 1.41,104 ± 1.87, and 112 ± 0.43 for the first, second, and third experimental groups, respectively). Although the second (KSGP 0240) and third (KSGP ARRIAH) groups did exhibit protection, they showed lower titers; nevertheless, they were still protective as homologous vaccines used in this study, and the differences in protection among the vaccines could be due to the origin of the vaccine and challenge strains used [21].

It should be noted that a more extensive sample range with a prolonged observational period would provide a significantly more detailed picture of the true potency of the evaluated vaccines.

## 5. Conclusions

In conclusion, despite the extensive successful application of the NISKHI ARRIAH vaccine in the post-Soviet Union space to curb SPPV, reports pertaining to its efficacy and potency are now becoming available to the broader research community. However, similar findings are lacking, hindering cross-strain comparison to see how they achieve an effective protective phenotype in a real field scenario. In the future, a comparison of such data, as well as the acquisition of additional experimental and field insights into vaccine efficacy and protective features, will allow the development of a comprehensive understanding of effective SPPV control and eradication in a given environment.

## Figures and Tables

**Table 1:** qPCR results detecting the SPPV genome in the blood and nasal swabs of the control group animals after challenge (Ct values).

		Day Post Infection							
№ Animal	Sample	1	2	3	5	7	9	12	14
21	Blood	N	N	N	34.00	N	N	N	N
	Swab	N	N	N	N	28.74	34.72	38.61	N
22	Blood	N	N	N	N	N	N	N	N
	Swab	N	N	N	N	24.20	34.55	38.38	N
23	Blood	N	N	N	N	27.62	28.66	28.97	35.50
	Swab	N	N	N	34.10	24.20	23.10	18.25	22.52
24	Blood	N	N	N	37.27	28.78	27.04	28.57	31.56
	Swab	N	N	N	28.81	22.07	18.54	18.68	21.90
25	Blood	N	N	N	35.26	26.43	28.08	Animal died	
	Swab	N	N	N	29.32	22.04	18.25		

N—negative qPCR result (genome not detected); Ct < 38—positive; Ct ≥ 38—doubtful.

## Data Availability

The original contributions presented in this study are included in the article. Further inquiries can be directed to the corresponding author.
